# The continuum of causality in human genetic disorders

**DOI:** 10.1186/s13059-016-1107-9

**Published:** 2016-11-17

**Authors:** Nicholas Katsanis

**Affiliations:** Center for Human Disease Modeling, Duke University Medical Center, Durham, NC 27701 USA

## Abstract

Studies of human genetic disorders have traditionally followed a reductionist paradigm. Traits are defined as Mendelian or complex based on family pedigree and population data, whereas alleles are deemed rare, common, benign, or deleterious based on their population frequencies. The availability of exome and genome data, as well as gene and allele discovery for various conditions, is beginning to challenge classic definitions of genetic causality. Here, I discuss recent advances in our understanding of the overlap between rare and complex diseases and the context-dependent effect of both rare and common alleles that underscores the need for revising the traditional categorizations of genetic traits.

## Introduction

**Fig. 1:**
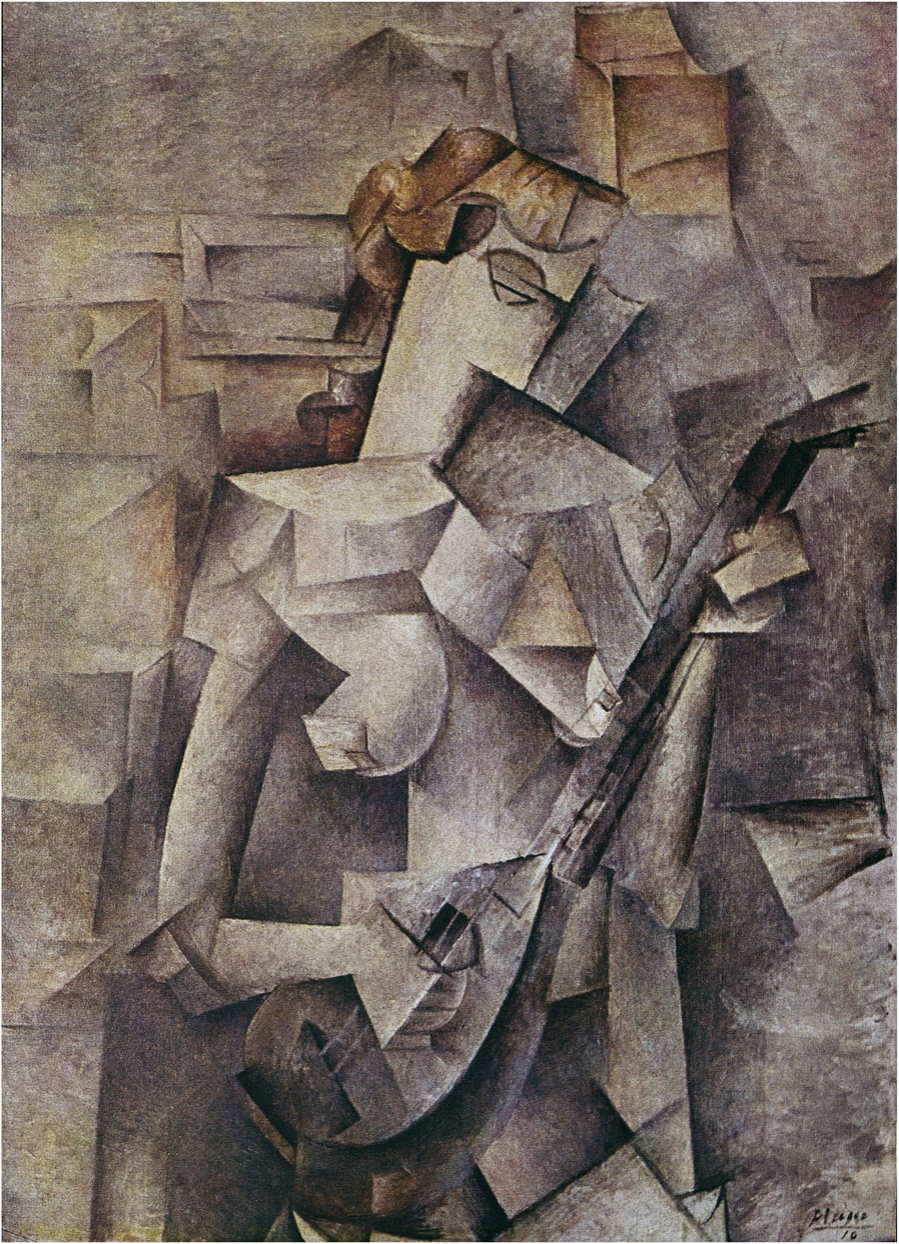
Girl with a Mandolin. © 1910 Estate of Pablo Picasso. Reproduced with permission, Artists Rights Society (ARS), New York, USA

At the start of the 20th century, a new post-modernist art movement, known as cubism, sought to deconstruct complex images into small geometrical shapes so that objects could be studied from multiple angles and viewpoints. An example of this type of art is Pablo Picasso’s Girl with a Mandolin (Fig. 1). Unbeknownst to the artists who propagated this aesthetic concept, the decomposition of complex problems into smaller, experimentally tractable parcels has in fact been a fundamental tenet of the scientific enterprise. In a cubist manner, complex multidimensional questions have been deconstructed, the ultimate aspiration being that once each compartment is understood we will be able to synthesize the complete image and gain clarity. Studies of human genetic disorders have traditionally, and faithfully, followed a reductionist paradigm. Human genetics typically separates rare and complex disorders into separate categories; has stratified the effect of alleles on the basis of their frequencies in populations; and has argued, passionately, over the contributions of rare versus common alleles in various disorders [1]. However, with the advent of data from the exomes and genomes of 10^5^–10^6^ people [2–5], including individuals diagnosed with particular disorders [6–10], it is clear that there is an imperfect fit between observation and traditional reductionist paradigms. For example, we have defined monogenic and polygenic traits on the basis of our perception of whether a phenotype is caused by mutations in one gene or many genes, with the ambiguous term “oligogenic” being using as an intermediate construct. Likewise, we have delineated an arbitrary cutoff of a 1% allele frequency to label an allele as “rare” (and 0.1% for “ultra-rare”), even though these definitions are both non-quantitative in the strict sense and are derived from imperfect observations in a subset of human populations.

## Challenging concepts in the study of monogenic human diseases

The foundational question of how a genotype influences a phenotype or multiple phenotypes has propagated the development of categories labeled with the approximate titles and imperfect terms mentioned above. This is not a failure of the field. Rather, it is a sign of maturity; a signal that it is time to reconsider how best to put together our first-order constructs to generate an accurate biological understanding of human diseases. Like all events that drive higher-order synthesis, challenges remain in the study of the genetics of human disease, not least because it will be necessary to discard some of our preconceived notions and to develop new language that is capable of capturing and transmitting more complex information efficiently.

Human genetics has, to date, exerted its greatest practical influence in the identification of genes and alleles that cause monogenic disorders. When considering the scope of current and planned projects [6–9], the ability to identify the “cause” of disease in most familial cases is likely to be limited. This is in part due to the fact that definitions of monogenic and polygenic disorders are becoming less distinct, which challenges existing concepts in the field of human disease genetics. At the same time, it is becoming increasingly clear that many of the conceptual aids that we have used to get to this point require reevaluation and revision.

We first consider perhaps the simplest concept: the concept of “one gene, one phenotype” [11]. We now understand that mutations in a single gene can drive numerous disorders in which the phenotype (or phenotypes) can be explained by the direction of a mutational effect (or mutational effects) at a single locus. Two examples of this are Kallman syndrome and Pfeiffer syndrome, both of which are characterized by distinct phenotypes that are caused by loss-of-function and gain-of-function mutations in *FGFR1* (which encodes fibroblast growth factor receptor 1), respectively [12]. The contribution of mutational effects to other so-called “monogenic” diseases is less clear. In such cases, environmental and/or genetic modifiers can influence the observed phenotypes. One such example is Fuchs corneal dystrophy, which is one of two autosomal-dominant disorders caused by mutations in *TCF4* (which encodes transcription factor 4); in this case, the disease can be defined as a non-penetrant Mendelian disorder or a complex trait [13], as modifier genes and/or environmental factors influence the observed phenotype [14]. For the discussed examples, a complex pattern of transcript splicing might explain the different phenotypes that are observed [15]. An extreme example might be the recessive loss-of-function mutations in *CEP290* (which encodes centrosomal protein 290) that cause a range of conditions, from the relatively mild disorders Leber congenital amaurosis or nephronophthisis to the perinatally lethal Meckel-Gruber syndrome [16–20]. Perhaps the best-known example is allelic variation in *CFTR* (which encodes cystic fibrosis transmembrane conductance regulator); the same *CFTR* mutation can cause a range of conditions, from isolated male infertility [21] to severe lung disease [22], probably owing to the influence of genomic context. These exemplars are unlikely to be academic curiosities; emerging reports from exome-based clinical genetic tests are reporting “phenotypic expansions”, which are defined as an increasing number of monogenic disorders that violate the “one gene, one phenotype” assumption. Presently, as many as ~25% of cases that have been exome sequenced in the clinical setting are being redefined [7]. Taking a cubist view, interpreting *CFTR* mutational data might be thought of as studying the pattern of a single cube, rather than looking at the relation of that cube to the whole “picture”.

Following on from the need to revise the concept of “one gene, one phenotype” is that of necessity and sufficiency. For much of the Mendelian disease era, alleles propagated in families or large multigenerational pedigrees have been described as both necessary and sufficient to cause disease; embedded in this concept is the notion that most alleles associated with Mendelian traits are penetrant. This concept is being challenged by the accrual of genomic data that are beginning to suggest that individuals vary in their tolerance of pathogenic mutations [23]; we are now aware of the presence of non-penetrant mutations in individuals with classically defined dominant or recessive traits [24]. Moreover, the traditional argument regarding the penetrance of Mendelian mutations may end up being circular, as the known alleles are those that were detected by available methods. The degree of phenotypic penetrance is influenced by stochastic forces, by imperfect phenotyping, sequencing, or annotation, and by the effect of genetic modifiers is yet to be determined. The degree to which phenotypic penetrance is influenced by stochastic forces, by imperfect phenotyping, sequencing, or annotation, and by the effect of genetic modifiers is yet to be determined.

Following on from the concept of necessity and sufficiency, a third concept that needs to be revised is the traditional paradigm that alleles associated with a rare disease are themselves rare in a population. This paradigm remains largely true, although the phenotypic effect (or effects) of some rare alleles is now known to be potentiated by common alleles. These rare alleles sometimes map to the disease locus, as exemplified by a common regulatory variant in *RET* (which encodes RET proto-oncogene) that contributes to Hirschsprung disease [25] and a promoter polymorphism in *FECH* (which encodes ferrochelatase) that regulates the penetrance of a rare mutation further downstream in the same gene [26]. More recently, the phenomenon of *cis*-complementation was described; this phenomenon describes how the deleteriousness of an allele can be modulated by neutral alleles in the same gene or haplotype [27]. In other instances, the common allele is not present in the “Mendelian gene” but in a discrete locus. For example, a common permissive microsatellite array at the *D4Z4* locus modulates the penetrance of mutations in *SMCHD1* (which encodes structural maintenance of chromosomes flexible hinge domain-containing protein 1) and causes facioscapulohumeral muscular dystrophy type 2 [28]. Similarly, an allele found in 3% of Europeans that potentiates an exonic splice enhancer in *CCDC28B* (which encodes coiled-coil domain-containing protein 28B) can modify the penetrance of a recurrent mutation in *BBS1* (which encodes Bardet-Biedl syndrome 1) in patients with Bardet-Biedl syndrome [29].

Imagining a cubist’s interpretation of this landscape might lead us to dissolve or blend some of the current “hardfixed” boundaries that are used to define monogenic disorders. Critically, it might be important to re-evaluate the usage of deterministic language, such as the terms “causes” or “solved”, as such terms simply and inaccurately imply that monogenic disorders are penetrant and minimally variable and that alleles at Mendelian loci cannot be influenced by the surrounding genome.

## Refining our understanding of complex human disorders

The traditional compartmentalization of complex disorders is also coming under scrutiny, and we are beginning to understand that the notion that drivers of common diseases are either exclusively common [30] or a collection of rare alleles [31] is an empirical oversimplification [32]. The idea that both common alleles of small effect and rare alleles of large effect are integral constituents of the genetic architecture of complex traits is largely accepted [33]. The purist cubist might argue that some complex traits are a cluster of rare disorders, whereas others are truly complex. For example, age-related macular degeneration is a paragon of complex trait analysis and became the first success story in the field of human disease genetics when genome-wide association studies found that a significant proportion of the genetic burden of disease was attributable to a common allele in *CFH* (which encodes complement factor H) [34–37]. However, rare alleles in a gene encoding another member of the complement pathway, *CFI* (which encodes complement factor I), have also been shown to be potent disease drivers [38], but they seem to behave in an almost Mendelian fashion because of their penetrance. In other complex traits, such as autism, the distinction between rare and common causative alleles is even more blurred; epidemiological and genomic studies have shown that most of the heritability of autism is due to common alleles, but penetrant de novo mutations can contribute substantially to an individual’s susceptibility to developing autism [39]. Post hoc examination of some of these rare, de novo alleles suggests that they have the capacity to cause syndromic phenotypes. An example of such an allele is the 16p11.2 deletion, which is found in >1% of cases of Autism spectrum disorder [40] and is associated with, for example, weight-regulation defects [41, 42], facial dysmorphisms and renal pathologies [43]. Similarly, re-examination of the mutational distribution of neurodevelopmental traits—including epileptic encephalopathy, intellectual disability, autism and schizophrenia—have shown extensive overlap [44]. In light of these findings, some might argue that some complex traits are a cluster of rare disorders, whereas others are truly complex. In the case of autism, for example, is it a constituent component of multiple rare syndromes or is it a continuum of variable expressivity and penetrance that does not fit neatly into either the Mendelian or the complex disease construct?

In some ways, cubist deconstruction will teach us that the traditional artificial boundaries are heuristically helpful but unnecessary, as key questions remain regarding the genetic variants that cause disease, the underlying molecular mechanisms of disease, and the direction of effect of variants associated with disease (i.e., whether they increase or decrease the expression or activity of the gene product). In this context, the rarity of some alleles and the strength of their effect on protein function have provided a bridge between rare and complex traits. For example, mutations in *MC4R* (which encodes melanocortin 4 receptor) cause a Mendelian form of severe obesity [45, 46] but have been proposed to predispose to adult-onset obesity under a complex trait model [47, 48]. Similarly, recessive mutations in *BBS10* (which encodes Bardet-Biedl syndrome 10)—mutations in which cause the rare Bardet-Biedl syndrome, a multisystemic disorder that also manifests truncal obesity [49]—have been found in individuals with morbid obesity and type 2 diabetes but no evidence of syndromic disease [50]. Although the presence of mutations in *MC4R* or *BBS10* in cohorts with adult-onset obesity informs us about the potential drivers of disease in only a miniscule number of individuals, they nonetheless teach us about two signaling cascades that are likely to be relevant to a larger proportion of patients. Similarly, although the contribution of *CHD8* (which encodes chromodomain helicase DNA binding protein 8) to autism will probably never exceed an infinitesimal proportion of the Autism spectrum disorder burden, understanding how the loss of CHD8 function affects neurodevelopment will be profoundly informative. Given the pace of allele discovery for both “monogenic” and “complex” traits, the discovery of alleles that provide causal evidence for particular loci, and those that establish the direction of effect, will increase dramatically and provide invaluable insights in terms of both biological understanding and drug discovery.

## Moving beyond traditional definitions of monogenic and polygenic human diseases

Using the analogy of cubist art, how then should we move forward and reconstruct the art piece “Girl with a Mandolin” (Fig. 1) so that we can appreciate all of her facets and beauty as a whole? That is, how can we develop a more accurate understanding of human genetic diseases by taking a cubist approach? For clinical diagnosis the emphasis on rare penetrant alleles must persist in order to understand causality and develop interventionist strategies; however, this must be coupled with improved statistical models for assessing the contribution of multiple factors, both genetic and non-genetic, to complex traits. In age-related macular degeneration, for example, the susceptibility of individuals who carry a combination of risk genotypes and smoke is sufficiently high to be clinically meaningful and behaviorally actionable [51], whereas for other disorders, such as type 2 diabetes, a scale and a tape measure remain more effective diagnostic tools than a genetics-based approach. If the question is one of therapeutics, however, then disease frequency becomes less relevant and allele causality, direction of effect, and biochemical cascades become key.

In both contexts, it will be important to start considering not just individual alleles or genes, but biological modules and pathways in toto. For example, considering biological modules has informed penetrance and expressivity of ciliopathies [52] and also illuminated the genetic architecture of peripheral neuropathies [53]. Likewise, for complex traits, considering genes that encode voltage-gated calcium ion channels as a group has revealed a causal module in schizophrenia that might eventually be druggable [54]. Indeed, one could conceive a pathway or a macromolecular complex as “the locus” and treat it as such, from both genetic and drug-discovery standpoints. Finally, we should not lose sight of the fact that the concept of “mutation” signifies nothing more than variation from a reference genome and in itself does not carry a detrimental connotation. In that context, identifying either rare or common variation that is deleterious to protein function but beneficial to an organism might provide unexpected and orthogonal avenues for therapeutic development, as exemplified by the protective effect of loss-of function mutations in *SLC30A8* (which encodes solute carrier family 30 member 8) to type 2 diabetes [55]. Welcome to the post-modernist era!

## Abbreviations

ASD: Autism spectrum disorder
*BBS1*: Bardet-Biedl syndrome 1
*BBS10*: Bardet-Biedl syndrome 10
*CCDC28B*: Coiled-coil domain-containing protein 28B
*CEP290*: Centrosomal protein 290
*CFH*: Complement factor H
*CFI*: Complement factor I
*CFTR*: Cystic fibrosis transmembrane conductance regulator
*CHD8*: Chromodomain helicase DNA binding protein 8
*FECH*: Ferrochelatase
*FGFR1*: Fibroblast growth factor receptor 1
*MC4R*: Melanocortin 4 receptor
*RET*: RET proto-oncogene
*SLC30A8*: Solute carrier family 30 member 8
*SMCHD1*: Structural maintenance of chromosomes flexible hinge domain-containing protein 1
*TCF4*: Transcription factor 4
